# Enzyme kinetics of deoxyuridine triphosphatase from Western corn rootworm

**DOI:** 10.1186/s13104-023-06618-2

**Published:** 2023-11-16

**Authors:** Carlos Riera-Ruiz, Hideaki Moriyama

**Affiliations:** 1 School of Biological Sciences, University of Nebraska–Lincoln, 243 Manter Hall, Lincoln, NE 68588-0118 USA; 2 Escuela Superior Politécnica del Litoral, Centro de Investigaciones Biotecnológicas del Ecuador, ESPOL Polytechnic University, ESPOL, Campus Gustavo Galindo Km 30.5 Vía Perimetral, P.O Box 09-01-5863, Guayaquil, Ecuador

**Keywords:** Western corn rootworm, *Diabrotica virgifera virgifera*, 5ʹ-triphosphate nucleotidohydrolase (dUTPase), dUTP, Thymidylate synthase, dUMP

## Abstract

**Objective:**

The Western corn rootworm (WCR), *Diabrotica virgifera virgifera*, is a highly adaptable insect pest that has evolved resistance to a variety of control strategies, including insecticides. Therefore, it is interesting to examine how housekeeping proteins in WCR have been changed under WCR-controlling strategies. In this study, we focused on one of such proteins in WCR, a ubiquitous enzyme 5'-triphosphate nucleotidohydrolase (dUTPase). In the thymidine synthetic pathway, dUTPase hydrolyzes deoxyuridine triphosphate (dUTP) and supplies the substrate, deoxyuridine monophosphate, for the thymidylate synthase (TS). It decreases the cellular content of uracil, reducing uracil misincorporation into DNA. Suppressing the dUTPase activity, therefore, contributes to thymineless death. In this study, we investigated the enzymatic properties of dUTPase.

**Results:**

The WCR *dUTPase* gene (*DUT*) was synthesized with the addition of His-tag corresponding DNA sequence and then cloned and expressed in *Escherichia coli*, and the protein product was purified. The product of WCR DUT hydrolyzed dUTP and was designated as dUTPase. WCR dUTPase did not hydrolyze dATP, dTTP, dCTP, or dGTP. WCR dUTPase was analyzed via size-exclusion chromatography and exhibited a molecular weight corresponding to that of trimer. The present format can be interpreted as nuclear trimer type. Possible isomers will be examined once transcriptome analyses are conducted.

**Supplementary Information:**

The online version contains supplementary material available at 10.1186/s13104-023-06618-2.

## Introduction

*Diabrotica virgifera virgifera* LeConte, commonly known as Western corn rootworm (WCR), is a major corn pest in North America [1, 2]. WCR is highly adaptable and has evolved resistance to a variety of management strategies, including crop rotation, synthetic insecticides, and genetically modified corn expressing Cry proteins [3]. To understand the mechanisms and effects of acquiring such resistance, it is important to determine whether the functions of any housekeeping proteins have been affected in WCR during the resistance evolution.

**Fig. 1:**
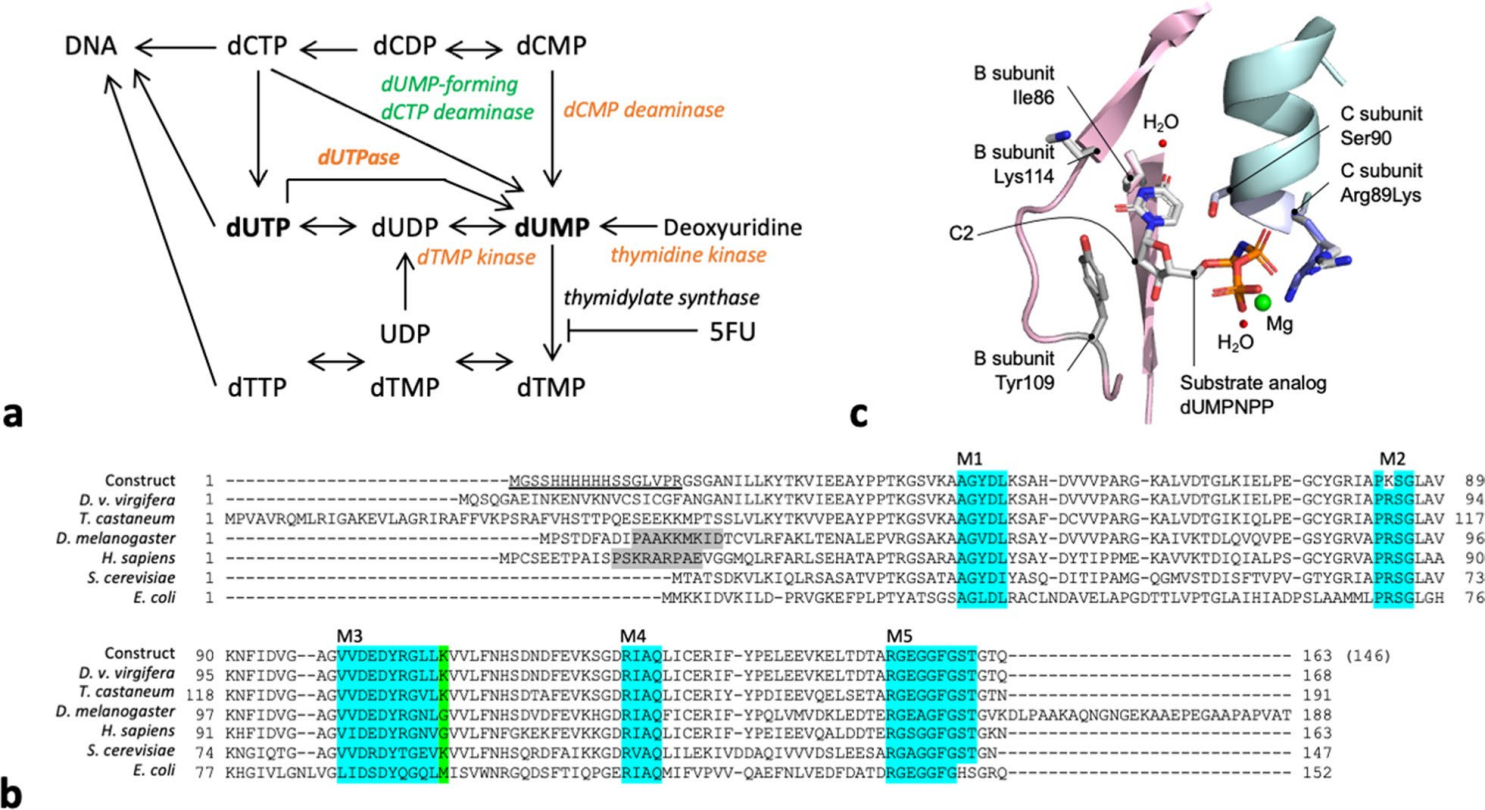
*DUT* gene from Western corn rootworm. **a** Pyrimidine metabolism map adapted from KEGG map00240 [7]. Enzymes supplying dUMP that are identified in WCR and *Methanococci* are indicated in orange and green, respectively. **b** Alignment of the dUTPase protein sequences among *D. v. virgifera*, *T. castaneum*, *D. melanogaster*, *H. sapiens*, *S. cerevisiae*, and *E. coli* (see Materials and Methods for the accession numbers). The construct sequence used in this study is shown at the top, where the positions including the removable N-terminal 6-His tag and the thrombin cleavage sites are underlined. The five conserved motifs in dUTPase are highlighted in cyan (M1–M5; see also Additional file 1: Fig. S1a). Substituted amino acids within the conserved motifs are not highlighted. Nuclear localization signals reported in *D. melanogaster* [25] and *H. sapiens* [15] are highlighted in gray. dUTPase from *D. melanogaster* possesses a *Drosophila*-specific 28-residue segment at the C-terminal [25]. The location of Lys114 in *D. v. virgifera* and corresponding residues in other species are highlighted in green. **c** Model for one of the active sites (Additional file 1: Fig. S1b). A substrate and side chains of relevant amino acid were shown in stick model

The ubiquitous and essential housekeeping enzyme deoxyuridine 5'-triphosphate nucleotidohydrolase (dUTPase) is involved in thymine synthesis (Fig. 1a) [4–7]. dUTPase removes pyrophosphate from deoxyuridine triphosphate (dUTP) to produce deoxyuridine monophosphate (dUMP), which is the substrate for thymidylate synthase (TS) [8, 9]. TS then methylates dUMP to produce dTMP [10, 11]. dUTPase, therefore, plays two critical roles: production of dUMP, the deoxythymidine triphosphate (dTTP) precursor, and degradation of dUTP to dUMP, preventing uracil misincorporation into DNA. Thymine is required in tissues with active DNA synthesis. Therefore, the thymidylate synthesis pathway, for example, is the point of action for several anticancer agents in humans [12–15]. However, redundancy of the supply of the substrate dUMP often limits the anticancer effect of pyrimidine antagonists [16, 17]. 5-Fluorouracil (5FU) is a TS inhibitor and used for cancer therapy where a large amount of accumulated dUMP leads to uracil misincorporation [18, 19]. In planarians, administration of 5FU caused death from DNA fragmentation [20, 21].

Knockout of the dUTPase gene (*DUT*) in mice [22] and knockdown of *DUT* mRNA in planarian [20, 21] are shown to be fatal. In insects, *DUT* silencing efficiently killed fruit fly, *Drosophila melanogaster*, at the early pupal stage [23–25]. Furthermore, knockdown of *DUT* expression resulted in a 100% mortality in red flour beetle, *Tribolium castaneum*, at the larval stage [26]. For WCR, feeding of *DUT* dsRNA to neonates for 9 days killed 54% of the larvae and inhibited the growth of 80% of the survivors [26].

In this study, the WCR *DUT* gene was synthesized and dUTPase was produced in *E. coli* and purified. Furthermore, the quaternary structure and enzymatic kinetics of WCR dUTPase were analyzed.

## Materials and methods

This study was conducted under the oversight of the Institutional Biosafety Committee at the University of Nebraska–Lincoln, Protocol Number 174.

### dUTPase genes and proteins

Genomic sequence (GenBank, NW_021039130.1) of *Diabrotica virgifera virgifera* LeConte (WCR) contained dUTPase gene (DUT; mRNA, XM_028280744.1; protein, XP_028136545). The dUTPase protein sequences used in this study are *Tribolium castaneum* (EFA05862.1), *Drosophila melanogaster* (Q9V3I1), *Saccharomyces cerevisiae* (P33317), *Homo sapiens* (P33316), and *E. coli* (strain K12; P06968).

### WCR *DUT* gene construct

For the construct design, SWISS-MODEL [27–30] and ProtParam [31] were used for molecular modeling and protein parameter calculation, respectively (Additional file 1: Fig. S1). A DNA fragment encoding WCR dUTPase was synthesized as follows. An N-terminal 6-His tag was introduced to enable metal affinity chromatography purification (Fig. 1b, shown as “construct”). The construct started at the 25^th^ residue of the WCR dUTPase to maintain the ability of the subunits to assemble in trimers based on sequence comparisons and 3D structural modeling (Additional file 1: Fig. S1ab and 1c). To prevent thrombin from cleaving other than the 6-His tag (secondary cleavage), the 89th arginine (R) residue, which is located in a helix and slightly away from the surface, was substituted to a lysine (K) with a G/A mutation (Fig. 1b and Additional file 1: Fig. S1b). Although the Arg89 plays a role in holding substrates, metals, and waters, lysine89 should be able to retain this function as it also has a positive charge. Arg89Lys mutation has also been shown to maintain the activity in the planarian dUTPase [20]. After optimizing the codon utilization for *E. coli* genes, a DNA fragment encoding the WCR DUT was synthesized by Gene Script (Piscataway, NJ, USA; Additional file 2: Fig. S2). The DUT gene was cloned into pET-15 (Novagen, Madison, WI, USA) using the NcoI and XhoI sites.

### Production of dUTPase and size analysis

**Fig. 2:**
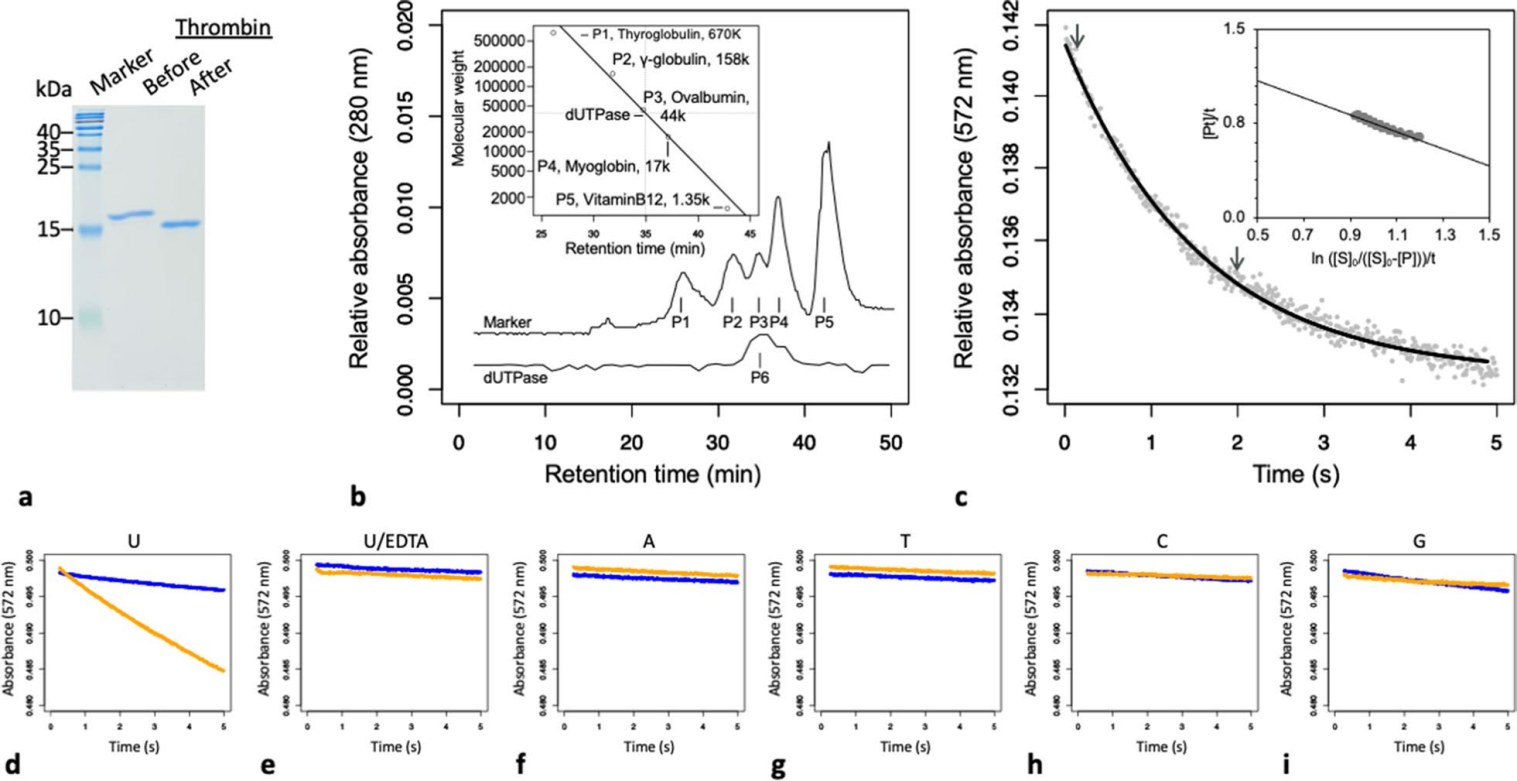
Purification, quaternary structure, and enzyme activities of WCR dUTPase. **a** 18% SDS-PAGE. The molecular weights of 6-histidine-tagged dUTPase and dUTPase after being cleaved by thrombin are 17.5 and 15.6 kDa, respectively. **b** Size-exclusion chromatography. The estimated molecular weight of tag-free dUTPase was 48 kDa. **c** Hydrolysis of dUTP by dUTPase. Gray dots indicate the observed drop in absorbance and the predicted regression line obtained from a corresponding scan. The inset indicates linear transformation of the data between the arrows according to the integrated Michaelis–Menten equation and the corresponding regression line. **d–i.** Substrate specificity of WCR dUTPase. In each plot, the orange and blue lines show when a particular substrate is added and no enzyme is added, respectively. The substrates used are dUTP (**d** and **e**), dATP (**f**). dTTP (**g**), dCTP (**h**), and dGTP (**i**). The enzyme is preincubated with 5-mM EDTA in e

The WCR dUTPase was prepared as previously described [32–34] (Fig. 2). After the chromatographies using Ni–NTA Resin (New England BioLabs, Ipswich, MA, USA) and then HiTrap Q (GE HealthCare, Chicago, IL, USA), approximately 10 mg of WCR dUTPase was purified from 2.5 g of cells. To remove the His-tag, tagged dUTPase was treated with human α-thrombin (Hematologic Technologies, Essex Junction, VT, USA) after dialysis against 20-mM Tris–HCl and 100-mM NaCl (pH 8). His-tagged proteins were removed by the Ni–NTA column, and protein was purified using Benzamidine Sepharose (GE HealthCare). Proteinase inhibitors were used, including 1-mM PMSF and 0.1-mg/mL benzamidine. Tryptic digestion and mass spectrometry confirmed the produced molecules [35–38] (Additional file 3: Fig. S3). To estimate the molecular weight of WCR dUTPase, size-exclusion chromatography was performed using Superdex Matrix (GE HealthCare). The molecular weight was calibrated using the SEC standard (BioRad, Hercules, CA, USA).

### Enzyme activity assay

Kinetic assay of WCR dUTPase was conducted using the cresol red method according to Larsson et al*.* [39] (Figs. 2c–i) on a high-speed spectrophotometer (Hi-Tech SF-61DX2, TgK Scientific, Bradford-on-Avon, UK). Absorbance data from each run was fitted to the function:

1 $$y=ae^{-xb}+c$$

where *x* and *y* denote time and absorbance, respectively. The predicted points were used to calculate the concentration of product formation at time *t*, [*P*]_*t*_, using the following equation:

2 $${[P]}_{t}= \frac{{A}_{0}- {A}_{t}}{{A}_{0}- {A}_{\infty }}{[S]}_{0}$$

where [*S*]_0_ denotes the substrate concentration, and *A*_0_,* A*_*t*_, and *A*_∞_ denote absorbance at the start, time *t,* and end of the reaction, respectively. The time course reaction was calculated using the integrated Michaelis–Menten equation.

3 $$\frac{{[P]}_{t}}{t}=V- \frac{K}{t}\mathrm{ln}\frac{{[S]}_{0}}{{[S]}_{0}- {[P]}_{t}}$$

where *K* and *V* denote *K*_*M*_ and *V*_*max*_, respectively*.* Equations 2 and 3 were used to plot the graph of $\frac{{[P]}_{t}}{t}$ against $\frac{\mathrm{ln}\frac{{[S]}_{0}}{{[S]}_{0}-[{S]}_{t}}}{t}$. Linear regression was used to calculate the slope that gives the *K*_*M*_ for each experiment. *K*_*cat*_ and *K*_*cat*_/*K*_*M*_ were calculated using the propagation of error approach. Three experiments were performed under the same conditions and the average value was reported. All calculations were performed using R v3.5.3 (R Foundation, Vienna, Austria; /www.r-project.org).

## Results

### dUTPase production

There are monomeric, dimeric, and trimeric dUTPases [40]. The WCR dUTPase protein sequence was compared with the trimeric dUTPases (Fig. 1b). Five conserved motifs characteristic to the trimeric dUTPases (M1–M5) [40] were also found in WCR dUTPase (Figs. 1b and Additional file 1: Fig. S1a). We synthesized and cloned a DNA fragment encoding WCR dUTPase (shown as “construct” in Figs. 1b and Additional file 2: Fig. S2). After purification, SDS-PAGE revealed that the purified protein has a molecular weight of 15.6 kDa (Fig. 2a). Tryptic mass spectrometry confirmed the identity of the purified protein (Additional file 3: Fig. S3). A total of 32 fragments were exclusive to the amino acid sequence of WCR dUTPase. Size-exclusion chromatography revealed that the molecular weight of WCR dUTPase was 48 kDa (Fig. 2b). These observations indicate that WCR dUTPase forms a trimer, which is consistent with that indicated by sequence similarity and the five conserved motifs as described above.

### dUTPase activity

Cresol red assay [41] revealed that the purified dUTPase has enzymatic activity (Fig. 2c–d). The addition of EDTA at the final concentration of 0.5 mM prevented the color change of cresol red (Fig. 2e). We also observed no significant color change when the substrate was changed to either dATP, dTTP, dCTP, or dGTP (Fig. 2f–i). We recorded the reaction trace with enzyme and dUTP concentrations of 50 nM and 1 mM, respectively (Fig. 1c), and estimated K_M_ as 0.7 ± 0.1 μM (*p* < 0.01 for curve fitting and linear regression; Table 1).

**Table 1:** Kinetics of dUTPase of different species against dUTP

Species	k_M_ (μM)	*k*_*cat*_ (s^−1^)	*k*_*cat*_/k_*M*_ (M^−1^ s^−1^)	Source
*D. v. virgifera*	0.7 ± 0.1	30 ± 0.5	4 × 10^7^	This study
*D. melanogaster*	0.4	12	3 × 10^7^	[42]
*H. sapiens*	3.6 ± 1.9	6.7 ± 0.2	1.9 × 10^6^	[8]
*S.* cerevisiae	13.2 ± 0.6	9.6 ± 0.2	7.4 × 10^5^	[43]
*E. coli*	0.5	11	1.4 × 10^7^	[44]

*NR* Not reported by the authors

### Comparison of dUTPase activity between organisms

The K_M_ value for WCR dUTPase was estimated to be 0.7 μM, whereas those reported for other organisms were between 0.4 and 3.6 μM (Table 1). WCR dUTPase had a higher specificity constant (k_cat_/K_M_) than other eukaryotes. WCR dUTPase exhibited a strict preference for dUTP. However, human [8] and *D. melanogaster* dUTPases have exhibited slight activities against dTTP and dCTP [42, 45]. The multiple alignment and 3D structural modeling of WCR dUTPase showed that one of the amino acids within the M3 conserved motif, Lys114, is different from the corresponding amino acids in *D. melanogaster* and human dUTPases (Gly110 and Gly106, respectively; Fig. 1b). These conserved motifs are involved in the catalysis and interaction with the deoxyribose ring [43]. The presence of a Lys residue in the M3 motif can reduce the flexibility of the loop at this position due to possible additional hydrogen bonds. Thus, it potentially has a smaller chance of accepting a nucleotide other than dUTP.

The effect of sugar moiety on substrate specificity is evaluated as follows. In the dUTPase models from human (3ehw) and WCR, the C3–OH of 2'-deoxyribose forms a hydrogen bond with Asp102 (106 in WCR dUTPase) and C2 facing Tyr105 (109 in WCR dUTPase; Fig. 1c). This configuration seemed too narrow to accommodate C2–OH; repulsion due to pi and anion arrangement is also expected. In yeast dUTPase, the Tyr88Ala mutant enzyme with reduced steric hindrance has been reported to have equivalent reactivity toward both dUTP and UTP [46]. Side chains corresponding to Arg86, Asp87, Thr89, Glu91 in yeast dUTPase are toward outward from the active site. E. coli dUTPase has been reported to be active against UTP [47]. In M3, Val101 of human dUTPase (105Val in WCR dUTPase) is changed to Ile89 E. coli dUTPase. WCR dUTPase seemed to have difficulty accepting DUT as a substrate.

## Discussion

Previous knockdown of *DUT* expression was reported to result in 89%–100% mortality in *T. castaneum* larvae (via injection of 150 nL of 0.01–250-ng/μL dsDUT) [26]. In the same study, feeding assay with 500 ng/cm^2^ of dsDUT revealed only 54% mortality in WCR neonates at 9 days [26].

As illustrated in the metabolic pathway presented in Fig. 1a, four enzymes besides dUTPase (UniProt ID, A0A6P7FUX9_DIAVI) can produce dUMP (cf. KEGG map 00240). Among the four enzymes, potential WCR homologs were identified for dCMP deaminase (A0A6P7GGP3_DIAVI), dTMP kinase (A0A6P7FXJ0_DIAVI), and deoxyribonucleotide kinase (dNK, A0A6P7FA76_DIAVI). Those three enzymes were also observed in both *D. melanogaster* and *T. castaneum*. dUMP-Forming dCTP deaminase is observed in *Methanococci* but not in the three aforementioned insects. It is noteworthy that while mammals have two types of thymidine kinases (TK1-like and non-TK1-like [48]), insects have only one multisubstrate enzyme, dNK. Phylogenetic analyses revealed that insect dNK might have evolved from a more specialized TK2 (non-TK1-like) enzyme [49]. Thus, higher TK activity does not interfere with the redundant supply of dUMP described below.

The redundant dUMP supply may explain why suppression of dUTPase expression had limited effect on WCR mortality. Independent of the dUMP availability, administration of 5FU (KEGG, map00983), a TS inhibitor commonly used to treat cancer in humans [50], is expected to suppress thymine production (Fig. 1a). More investigations on dUTPase function, gene repertoire, and 5FU metabolism during the development of WCR and other coleopterans are warranted.

## Limitation

This study found that WCR DUT encodes a uracil-specific pyrophosphatase, which could explain the physiological effects of WCR DUT knockdown [26]. Future research is warranted to fully characterize WCR DUT.

The identification, confirmation, and characterization of possible isoforms of dUTPase are necessary to understand its structure and function. The levels of glycosylation and phosphorylation should also be considered, as these can affect the activity of the protein. Research on dUTPase in humans is ongoing, with the goal of developing new cancer chemotherapeutic agents and malaria treatments. Rácz et al*.* [51] have identified two additional isoforms of dUTPase in humans, DUT-N and DUT-M, which are localized to the nucleus and mitochondria, respectively. Future studies are warranted to investigate a molecular species that remains in the cytoplasm, which may represent a novel isoform of WCR dUTPase.

A study of dUTPase levels post-knockdown is key to understanding dUTPase inhibitors and pest control potential. TAS-114 [52] is a potent dUTPase inhibitor with chemotherapy applications. Although its inhibitory activity against WCR dUTPase has not been tested, it is hoped that similar environmentally friendly compounds will be developed.

### Supplementary Information

**Additional file 1: Fig. S1.** Structural modeling. **a **The monomer model of WCR dUTPase (subunit A). The five conserved motifs (M1-M5), Arg89, N- and C-termini are indicated. **b **The dUTPase trimer model. Chains B and C are added to the monomer view (a). The stick model represents the key residues in the active site. The boxed area is enlarged in Fig 1c. Method: Structural models were built using SWISS-MODEL [27] with a human dUTPase (PDB ID: 3ehw) as the template. Models for dUTPase from WCR in the native and mutated version, Arg89Lys, had QMEAN values of −0.3 and −0.35, respectively. Models are usable because the root mean square deviation between the model and template was less than 2 Å [28, 29] and the QMEAN was less than 1 [30]. Structural mining was performed using PyMOL (Version 2.0, Schrödinger, New York, NY, USA) and the ProtParam server was used to calculate protein parameters [31].

**Additional file 2: Fig. S2.** The WCR *DUT *construct sequence after being optimized for codon utilization of *E. coli*. The *DUT *construct sequence was cloned into pET-15 using *Nco*I and *Xho*I sites. One internal mutation (G → A at the 253th nucleotide) was placed to produce Arg89Lys. After thrombin cleavage (indicated by //), the N-terminal sequence, including the His tag, was removed.

**Additional file 3: Fig. S3.** Peptides identified by mass spectrophotometry. **a**. Mass spectrum. **b**. Identified peptides. The full-length of the dUTPase protein construct sequence was identified by MS/MS. Sixty-seven exclusive unique spectra were identified out of the 968 total spectra. Likely deaminated glutamines are highlighted in cyan. All 33 unique peptide fragments are aligned against the dUTPase construct sequence. The numbers after each aligned peptide fragment represent the absolute abundance out of the 968 spectra. Method: The SDS-band was cut [35, 36] and submitted for mass spectrometry at the University of Nebraska–Lincoln Proteomics and Metabolomic Research Core Facility (Lincoln, NE, USA) [37, 38].

## Data Availability

The datasets and plasmid DNA constructs used in this study are available from the corresponding author upon reasonable request.
