# Supplementary material for: Enzyme kinetics of deoxyuridine triphosphatase from Western corn rootworm
Source: BMC Res Notes. 2023 Nov 16;16:336. doi: 10.1186/s13104-023-06618-2 (PMC10652518; doi:10.1186/s13104-023-06618-2)
Supplement: Supplementary file 1 — Additional file 1: Fig. S1. Structural modeling. a The monomer model of WCR dUTPase (subunit A). The five conserved motifs (M1-M5), Arg89, N- and C-termini are indicated. b The dUTPase trimer model. Chains B and C are added to the monomer view (a). The stick model represents the key residues in the active site. The boxed area is enlarged in Fig 1c. Method: Structural models were built using SWISS-MODEL [27] with a human dUTPase (PDB ID: 3ehw) as the template. Models for dUTPase from WCR in the native and mutated version, Arg89Lys, had QMEAN values of −0.3 and −0.35, respectively. Models are usable because the root mean square deviation between the model and template was less than 2 Å [28, 29] and the QMEAN was less than 1 [30]. Structural mining was performed using PyMOL (Version 2.0, Schrödinger, New York, NY, USA) and the ProtParam server was used to calculate protein parameters [31]. [file 13104_2023_6618_MOESM1_ESM.pdf]

Supplemental materials

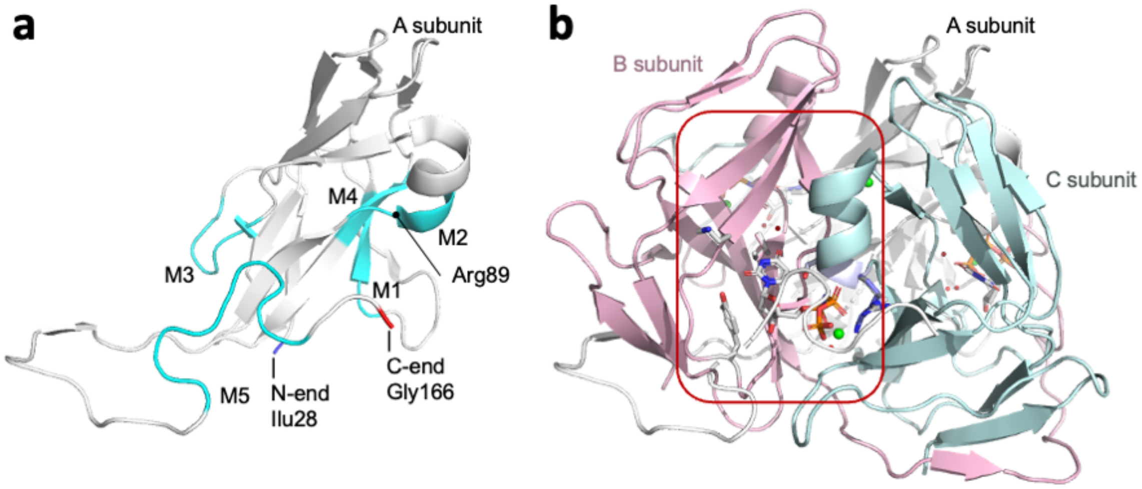

Fig. S1. Structural modeling. a. The monomer model of WCR dUTPase (subunit A). The five conserved motifs (M1-M5), Arg89, N- and C-termini are indicated. **b.** The dUTPase trimer model. Chains B and C are added to the monomer view (a). The stick model represents the key residues in the active site. The boxed area is enlarged in Fig 1c.

Method: Structural models were built using SWISS-MODEL [27] with a human dUTPase (PDB ID: 3ehw) as the template. Models for dUTPase from WCR in the native and mutated version, Arg89Lys, had QMEAN values of −0.3 and −0.35, respectively. Models are usable because the root mean square deviation between the model and template was less than 2 Å [28, 29] and the QMEAN was less than 1 [30]. Structural mining was performed using PyMOL (Version 2.0, Schrödinger, New York, NY, USA) and the ProtParam server was used to calculate protein parameters [31].
