# Supplementary material for: Enzyme kinetics of deoxyuridine triphosphatase from Western corn rootworm
Source: BMC Res Notes. 2023 Nov 16;16:336. doi: 10.1186/s13104-023-06618-2 (PMC10652518; doi:10.1186/s13104-023-06618-2)
Supplement: Supplementary file 2 — Additional file 2: Fig. S2. The WCR DUT construct sequence after being optimized for codon utilization of E. coli. The DUT construct sequence was cloned into pET-15 using NcoI and XhoI sites. One internal mutation (G → A at the 253th nucleotide) was placed to produce Arg89Lys. After thrombin cleavage (indicated by //), the N-terminal sequence, including the His tag, was removed. [file 13104_2023_6618_MOESM2_ESM.pdf]

Supplemental materials

```
NcoI   10         20         30         40         50         60
     CCATGGGCAG CAGCCATCAT CATCATCATC ACAGCAGCGG CCTGGTGCCG CGCGGCAGCG
       M  G  S   S  H  H   H  H  H  H   S  S  G   L  V  P   R//G  S  G
              70         80         90        100        110        120
     GAGCCAATAT TTTACTTAAA TACACCAAAG TAATTGAAGA AGCTTATCCT CCAACCAAGG
       A  N  I   L  L  K   Y  T  K  V   I  E  E   A  Y  P   P  T  K  G
             130        140        150        160        170        180
     GTTCTGTAAA AGCTGCTGGT TATGACTTAA AAAGTGCACA TGATGTCGTG GTACCGGCTA
       S  V  K   A  A  G   Y  D  L  K   S  A  H   D  V  V   V  P  A  R
             190        200        210        220        230        240
     GGGGCAAAGC CCTGGTGGAT ACAGGGCTGA AAATTGAACT ACCAGAAGGT TGCTATGGAA
       G  K  A   L  V  D   T  G  L  K   I  E  L   P  E  G   C  Y  G  R
             250        260        270        280        290        300
     GAATTGCTCC AAAATCTGGT TTAGCTGTAA AGAACTTCAT TGATGTCGGA GCTGGAGTAG
       I  A  P   K  S  G   L  A  V  K   N  F  I   D  V  G   A  G  V  V
             310        320        330        340        350        360
     TTGATGAAGA TTACCGTGGC CTACTGAAGG TTGTTCTATT CAACCATTCC GACAATGATT
       D  E  D   Y  R  G   L  L  K  V   V  L  F   N  H  S   D  N  D  F
             370        380        390        400        410        420
     TTGAAGTAAA GAGTGGAGAT AGAATTGCTC AGCTGATCTG TGAAAGAATC TTTTATCCTG
       E  V  K   S  G  D   R  I  A  Q   L  I  C   E  R  I   F  Y  P  E
             430        440        450        460        470        480
     AACTTGAAGA AGTAAAGGAG TTGACTGATA CTGCCCGTGG TGAAGGAGGC TTTGGATCCA
       L  E  E   V  K  E   L  T  D  T   A  R  G   E  G  G   F  G  S  T
             490        500
     CAGGAACCCA GTAACTCGAG
       G  T  Q   *    XhoI
```

Fig. S2. The WCR *DUT* construct sequence after being optimized for codon utilization of *E. coli*. The *DUT* construct sequence was cloned into pET-15 using *Nco*I and *Xho*I sites. One internal mutation (G → A at the 253th nucleotide) was placed to produce Arg89Lys. After thrombin cleavage (indicated by //), the N-terminal sequence, including the His tag, was removed.
