# Supplementary material for: Enzyme kinetics of deoxyuridine triphosphatase from Western corn rootworm
Source: BMC Res Notes. 2023 Nov 16;16:336. doi: 10.1186/s13104-023-06618-2 (PMC10652518; doi:10.1186/s13104-023-06618-2)
Supplement: Supplementary file 3 — Additional file 3: Fig. S3. Peptides identified by mass spectrophotometry. a. Mass spectrum. b. Identified peptides. The full-length of the dUTPase protein construct sequence was identified by MS/MS. Sixty-seven exclusive unique spectra were identified out of the 968 total spectra. Likely deaminated glutamines are highlighted in cyan. All 33 unique peptide fragments are aligned against the dUTPase construct sequence. The numbers after each aligned peptide fragment represent the absolute abundance out of the 968 spectra. Method: The SDS-band was cut [35, 36] and submitted for mass spectrometry at the University of Nebraska–Lincoln Proteomics and Metabolomic Research Core Facility (Lincoln, NE, USA) [37, 38]. [file 13104_2023_6618_MOESM3_ESM.pdf]

Supplemental materials

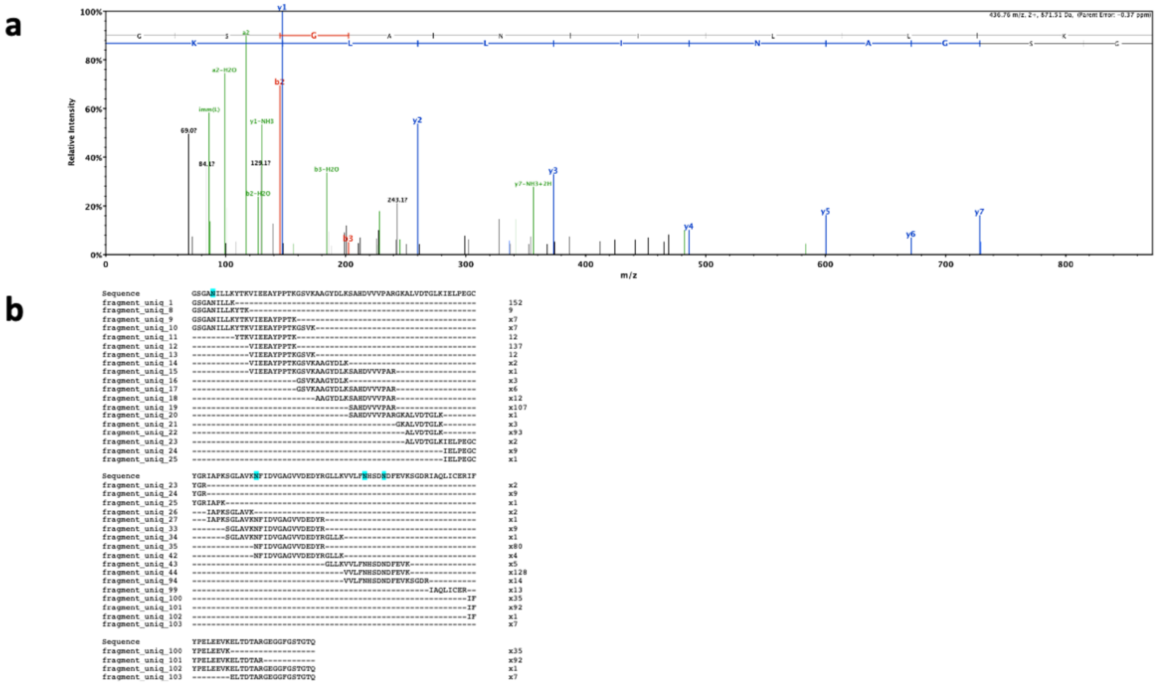

Fig. S3. Peptides identified by mass spectrophotometry. a. Mass spectrum. **b.** Identified peptides. The full-length of the dUTPase protein construct sequence was identified by MS/MS. Sixty-seven exclusive unique spectra were identified out of the 968 total spectra. Likely deaminated glutamines are highlighted in cyan. All 33 unique peptide fragments are aligned against the dUTPase construct sequence. The numbers after each aligned peptide fragment represent the absolute abundance out of the 968 spectra.

Method: The SDS-band was cut [35, 36] and submitted for mass spectrometry at the University of Nebraska–Lincoln Proteomics and Metabolomic Research Core Facility (Lincoln, NE, USA) [37, 38].
